# p5 Peptide-Loaded Human Adipose-Derived Mesenchymal Stem Cells Promote Neurological Recovery After Focal Cerebral Ischemia in a Rat Model

**DOI:** 10.1007/s12975-020-00805-0

**Published:** 2020-05-06

**Authors:** Arjun Paudyal, Flavia Semida Ghinea, Mircea Popescu Driga, Wen-Hui Fang, Giulio Alessandri, Laura Combes, Hans Degens, Mark Slevin, Dirk M. Hermann, Aurel Popa-Wagner

**Affiliations:** 1 Department of Life Sciences, Faculty of Science and Engineering, Manchester Metropolitan University, Chester Street, Manchester, UK; 2 Department of Human Movement Sciences, Faculty of Behavioural and Movement Sciences, Vrije University Amsterdam, Amsterdam Movement Sciences, Amsterdam, The Netherlands; 3 Doctoral School, Department of Center of Clinical and Experimental Medicine, University of Medicine and Pharmacy Craiova, Craiova, Romania; 4 Cellular Neurobiology Laboratory, Department of Cerebrovascular Diseases, IRCCS Neurological Institute C. Besta, 20133 Milan, Italy; 5 University of Medicine and Pharmacy, Targu Mures, Romania; 6 Lithuanian Sports University, Kaunas, Lithuania; 7 Institute of Dementia and Neurological Aging, Weifang Medical University, Weifang, China; 8 Department of Neurology Chair of Vascular Neurology and Dementia, University of Medicine Essen, Essen, Germany; 9 Griffith University Menzies Health Institute of Queensland, Gold Coast Campus, Gold Coast Campus, QLD 4222 Australia

**Keywords:** Cyclin-dependent kinase 5, Peptide 5, Neuroprotection, Human adipose-derived mesenchymal stem cells, Cerebral ischemia, Rat

## Abstract

Adipose-derived mesenchymal stem cells markedly attenuated brain infarct size and improved neurological function in rats. The mechanisms for neuronal cell death have previously been defined in stress states to suggest that an influx of calcium ions into the neurons activates calpain cleavage of p35 into p25 forming a hyperactive complex that induces cell death. Now we report that p5, a 24-residue peptide derived from p35, offers protection to neurons and endothelial cells in vitro. In vivo administration of human adipose-derived mesenchymal stem cells (hADMSCs) loaded with this therapeutic peptide to post-stroke rats had no effect on the infarct volume. Nevertheless, the treatment led to improvement in functional recovery in spatial learning and memory (water maze), bilateral coordination and sensorimotor function (rotating pole), and asymmetry of forelimb usage (cylinder test). However, the treatment may not impact on cutaneous sensitivity (adhesive tape removal test). In addition, the double immunofluorescence with human cell-specific antibodies revealed that the number of surviving transplanted cells was higher in the peri-infarcted area of animals treated with hADMSCs + P5 than that in hADMSC-treated or control animals, concomitant with reduced number of phagocytic, annexin3-positive cells in the peri-infarcted region. However, the combination therapy did not increase the vascular density in the peri-infarcted area after stroke. In conclusion, administration of hADMSC-loaded p5 peptide to post-stroke rats created conditions that supported survival of drug-loaded hADMSCs after cerebral ischemia, suggesting its therapeutic potential in patients with stroke.

**Electronic supplementary material:**

The online version of this article (10.1007/s12975-020-00805-0) contains supplementary material, which is available to authorized users.

## Introduction

Therapeutic standard procedure for ischemic stroke is cerebral artery recanalization with tissue-type plasminogen activator (tPA). However, brain inflammation and comorbidities have a negative impact on successful revascularization and functional outcome [1–5].

Numerous studies have shown that bone marrow mesenchymal stem cell (BMSC) transplantation could foster the recovery of neurological function and reduce infarct size in rats with acute ischemic stroke [6, 7]. In the animal model of cerebral ischemia, BMSC transplantation could improve neurological functional outcomes and decrease the infarction size via differentiation, replacement, and neural circuit reconstruction by enhancing angiogenesis [8]. Further, adipose-derived mesenchymal stem cells markedly attenuated brain infarct size and improved neurological function in rats [9].

Combinations of MSCs and the peptide granulocyte colony-stimulating factor (G-CSF), given separately, to post-stroke aged rats led to a robust and consistent improvement of neurological function after 28 days [10]. The combination therapy also led to robust angiogenesis in the formerly infarct core and in islets of the soft tissue beyond [11].

Cyclin-dependent kinase 5 (CDK5), a serine/threonine kinase, in complex with its activators, p35 (protein of 35 kDa) and p39 (protein of 39 kDa), is essential for early neurodevelopment in mammals [12]. However, a variety of neurotoxic conditions, such as ischemic brain damage, oxidative stress, amyloid-β peptide (Aβ), excitotoxicity, calcium dyshomeostasis, and inflammation, induce influx of Ca^2+^ ions and the rise in the intracellular Ca^2+^, thereby promoting activation of calpain, a Ca^2+^-activated protease, which in turn cleaves p35 into p25 and a p10 fragment [13, 14]. p25 forms a more stable CDK5-p25 hyperactive complex, which causes aberrant hyperphosphorylation of various substrates of CDK5 like tau and neurofilament, that leads to neuronal apoptosis and is associated with neuropathology. Therefore, a therapeutic approach directed specifically at CDK5-p25 complex might prove successful. To improve its therapeutic efficacy, several peptides consisting of amino acid residues of p35, such as CDK5 inhibitory peptide (CIP, a peptide of 125 amino acid residues), p10, and p5, have been generated and proven to specifically reduce CDK5-p25 increased activity without affecting the normal endogenous CDK5-p35 or other CDK activities [15–21]. In particular, p5 also reduced neuronal apoptosis induced by Ca^2+^ in hypoxia/ischemia brain injury [22, 23].

Recently, BMSCs have been used as vectors in cancer therapy by delivering oncolytic viruses and drug-loaded nanoparticles [15]. Moreover, a recent study has shown that MSC given through their natural container (MFAT) led to an improved MSC survival and therefore may prolong the delivery of the molecule/proteins transported [16]. However, the use of BMSCs as drug carrier of the CIP peptide for stroke has not been tested. In this work, we hypothesized that a prolonged delivery of the CIP peptide by fat-derived MSC may lead to improved post-stroke recovery by blocking neuronal and endothelial cell apoptosis through CDK5-p35 and subsequent p53 activation [17]. This pathway was previously shown to be a key initiator of cellular apoptosis following stroke, associated with excitotoxic release of calpains and subsequent hyper-phosphorylation of the CDK5 protein following p35 conversion to p25 [18, 19].

## Methods

### Cell Culture

Human adipose-derived mesenchymal stem cells (hADMSCs) were provided by Professor Giulio Alessandri and Professor Valentina Ceserani. hADMSCs were isolated from peri-umbilical fat tissue and characterized as described [20]. Human neuroblastoma neuroblastic type SH-SY5Y cells and bovine aortic endothelial cells (BAECs) were cultured in Dulbecco’s modified Eagle’s medium (DMEM; Lonza) supplemented with 10% FBS and 1% L-glutamine.

### p5 Priming of hADMSCs

p5, a 24-residue peptide, derived from p35, the CDK5 activator, was chemically synthesized with 5-FAM conjugated on the N-terminus and KRKR-KRKR wrapper on the C-terminus [15] (Cambridge Research Biochemicals). The sequence of p5 peptide is [5-FAM]-KEAF-W-DRCLSVINLMSSKMLQINAKRKRKRKR-amide. The toxicity of p5 on hADMSCs was determined in a 24-h alamarBlue assay (cytotoxicity test; Life Technologies) and in a 3-day alamarBlue assay (anti-proliferative test). Based on these results, the p5 priming of hADMSCs was carried out with two concentrations, 25 μg/ml and 100 μg/ml.

### In Vitro Proliferation Assay

The protective effect of both p5 or CM of p5 primed hADMSCs on SH-SY5Y cells and BAEC proliferation after the treatment of Ca^2+^ ionophore combined with CaCl_2_ was determined by the alamarBlue assay (Life Technologies). Briefly, 7.0 × 10^4^ SH-SY5Y cells or 1.0 × 10^3^ BAECs were plated in 96-well multiwell plates in DMEM. The cells were then treated with 5 μM calcium ionophore A23187 (Sigma-Aldrich) and 2.5 mM CaCl_2_ (Sigma-Aldrich) with or without p5 or CM of p5-primed hADMSCs. Three wells were devoted for the assay every day for 3 days, and time course of cell proliferation was monitored.

### Animals and Experimental Design

The subjects of these experiments were male Sprague-Dawley rats (*n =* 40; 3–4 months of age) 320–400 g kept under standard laboratory conditions with free access to food and water. The numbers reported in the results refer to the number of animals that survived the surgery and completed the 4-week testing period. All appropriate measures were taken to minimize pain and suffering.

### Randomization

A scientist was in charge of randomization by (i) group assignment; (ii) behavioral testing; (iii) surgery assignment; and (iv) treatment assignment.

### Behavioral Testing

To evaluate changes in neurological function associated with ischemia, the rats were subjected to a variety of somatosensory, motor, learning, and memory tests before and after surgery. All testing was performed from 09:00 to 11:00. Results obtained before surgery were used to define 100% functionality for each animal on each test, and functional recovery was expressed as percent recovery relative to the pre-surgery baseline.

### Bilateral Sensorimotor Coordination: Rotating Beam-Walking Test

The rotating pole task assesses coordination and sensorimotor function in the middle cerebral artery occlusion (MCAO) model. Each rat was tested for its ability to cross a rotating (6 rpm) horizontal rod. The score assessment was done as previously described [10, 21]. Briefly, the time taken for the rat to traverse the rotating cylinder and join a group of rats visible at the finish line was measured. The score assessment was twofold: (i) time (s) required to traverse the rotating cylinder and (ii) the score as follows: 0 = rat falls immediately (onto a soft surface); 1 = rat does not walk forward, but stays on the rotarod; 2 = rat walks, but falls before reaching the goal; 3 = rat traverses the rod successfully, but the limbs are used asymmetrically; 4 = the left hindlimb is used less than 50% of the time taken to traverse the rod; 5 = the rat successfully traverses the rod, but with some difficulties; 6 = no mistakes, symmetric movements.

### Asymmetric Sensorimotor Deficit: Adhesive Tape Removal Test (Tape Test) and Cylinder Test

We assessed the asymmetry of sensorimotor deficit of the forelimbs induced by unilateral MCAO, by tape test and cylinder test as previously described [10, 21]. For the cylinder test, the forelimb use asymmetry during vertical exploration of the walls of a 20-cm-diameter and 40-cm-height glass cylinder was measured. Rats which did not make at least 20 exploratory contacts with the walls were excluded from the analysis procedure. The asymmetry index was calculated as (Right − Left)/(Right + Left), where Right and Left are the number of contacts of the right.

### Spatial Learning and Memory: Morris Water Maze

The Morris water maze task was used to assess spatial learning and memory. One week before surgery, aged rats were trained to find a submerged platform in a large (180-cm-diameter) pool filled to within 20 cm of the upper edge with water maintained at 26 °C as previously described [10]. The swim path was recorded by an image analysis system (Noldus, Holland) that computed path length and percentage of time spent in each of the four quadrants.

### Surgery

Cerebral infarction was induced by transcranial interruption of blood flow by transiently lifting the middle cerebral artery (MCA) with a tungsten hook as previously described [19]. Throughout surgery, anesthesia was maintained by spontaneous inhalation of 2% isoflurane in a mixture of 70% nitrous oxide and 30% oxygen. After 90 min, the tungsten hook was released and the common carotid arteries were re-opened. Surgery was performed under antiseptic conditions to minimize the risk of infection. The rats then received 0.05 mg/kg SC buprenorphine (sc) for treatment of post-operative pain.

Subsequent to survival times of 28 days, the rats were deeply anesthetized and perfused with neutral buffered saline followed by buffered 4% freshly depolymerized paraformaldehyde. The brain was removed, post-fixed in 4% buffered paraformaldehyde for 24 h, cryoprotected in 15% glycerol prepared in 10 mM phosphate-buffered saline, flash-frozen in isopentane, and stored at − 70 °C until sectioning.

### Treatments

The contents of the frozen vials containing hADMSCs are re-suspended in a T75 flask containing 10 ml of stem cell medium (SCM). After a period of about 24 h, the media was removed and replaced with fresh SCM primed with the therapeutic peptide p5 at a final concentration of 10 μg/ml. After an incubation period of 24 h, the cells were washed 3 times with PBS before trypsinization. One group of rats (*n* = 8) was treated with 1 **×** 10^6^ hADMSCs, given intracortically at 3 different locations in the exposed area proximal to the occluded middle cerebral artery, immediately after stroke. The second group (*n* = 8) was treated with hADMSCs + p5 and the control (*n* = 8) group was treated with the vehicle.

### Determination of Infarct Volume by Immunohistostaining

To assess the size of the infarct induced by focal ischemia, we used mouse anti-NeuN immunostaining [10]. Every 20th free-floating section of 25 μm was immunostained for NeuN to cover the entire infarcted volume, which was then calculated as the sum of the partial areas using ImageJ.

### Collagen IV Immunofluorescence

Sections (25-μm-thick) were cut on a freezing microtome and processed for immunohistochemistry as previously described [10]. Briefly, tissue sections were exposed overnight at 4 °C to rabbit anti-collagen IV (1:2000, Abcam, UK) and the signal was amplified utilizing an anti-rabbit polymer-based secondary detection system (Histofine polymer-HRP, Nichirei, Japan) diluted 1:10 in PBS containing 1% normal goat serum and 0.3% Tween 20.

### HuNu/CD105 Double Immunofluorescence

For phenotyping, the tissue was incubated with a mix of rabbit anti-mouse HuNu (1:1000, Novus Biologicals, UK) and mouse anti-human CD105 (1:1000, antibodies-on-line, Aachen, Germany) at 4 °C overnight. Next day, sections were incubated with a mix of Alexa Fluor® 488 goat anti-rabbit IgG and Alexa Fluor® 555 goat anti-mouse IgG for CD105.

### Annexin A3/NeuN/IbaI Triple Immunofluorescence

Cryostat, free-floating sections of 25 μm were fixed in 4% paraformaldehyde for 15 min and then washed extensively with PBS and incubated serially with the following primary antibodies, rabbit anti-AnxA3 (1:500, Abcam, UK), goat anti-IbaI (1:3000, Abcam, UK), and mouse anti-NeuN (1:1000, Millipore, Germany). Secondary reagents were donkey anti-mouse Alexa 488, donkey anti rabbit-Alexa 568, and donkey anti goat-Alexa 647.

### Cell Counting of Co-localized Cells

The number of co-labeled cells (HuNu + CD105-positive) and (AnxA3/IbaI) cells at the reperfusion times of 28 days was determined by counting cells on every tenth section in systematic random series across the entire infarcted volume as previously described [10].

### Quantitation of Microvascular Density

Microvascular density was quantitated using the “hot spot” analysis. Briefly, hot spots, i.e., regions with a high density of collagen IV-positive microvessels, were identified using a × 40 objective and were then counted using × 20 objective, corresponding to a microscopic field of 0.7386 mm^2^ as previously described [11].

### Statistical Analyses

Statistical analysis was performed using SPSS (V 22.0). Behavioral data were analyzed using two-way ANOVA (treatment × duration) with post-stroke time as a repeated measurement factor. The Greenhouse-Geisser correction was applied where the sphericity was violated. *T* tests were performed for the single parameter comparison, and unequal variance between groups was assumed. All values are presented as mean ± SEM. *p* values < 0.05 were considered statistically significant.

## Results

### p5-Primed HADMSCs Protected Neurons and Endothelial Cells from Calpain-Induced Toxicity

**Fig. 1:**
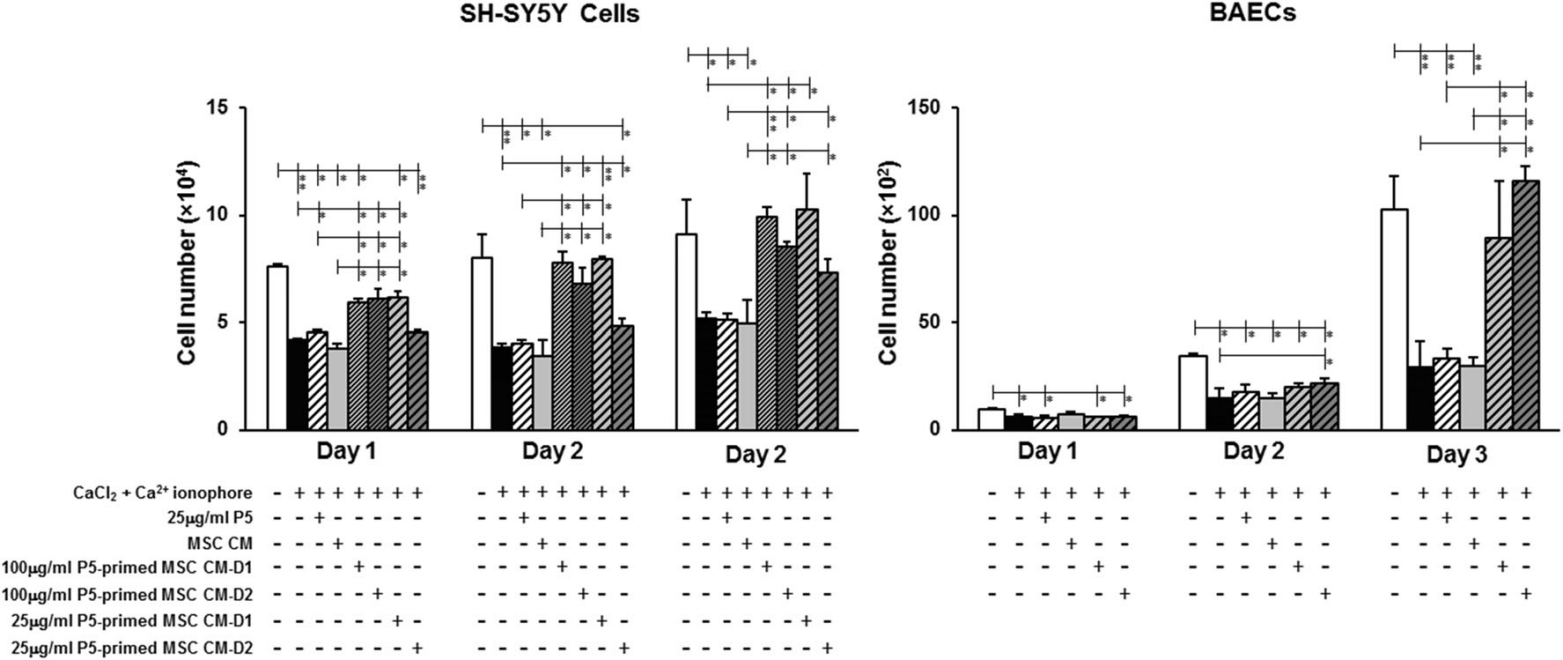
The conditioned medium (CM) from p5-primed hADMSCs protected human neurons (SH-SY5Y cells) and endothelial cells (BAECs) from calpain-induced cytotoxicity. The cultured SH-SY5Y cells and BAECs were treated with p5 or CM from 100 μg/ml or 25 μg/ml p5-primed hADMSCs collected on day 1 and day 2 for 1 h, then further treated with 5 μM calcium ionophore A23187 and 2.5 mM CaCl_2_ for 10 min. The cell proliferation was monitored for 3 consecutive days using the alamarBlue assay. Data are means ± SD (error bars). Significance (denoted as an asterisk) was calculated compared to the control using one-way ANOVA with Bonferroni post-test analysis (with *p < 0.05; **p < 0.01, 푛 = 3)

As the Ca^2+^ ionophore and CaCl_2_ treatment activated apoptosis-related genes, such as p53 and caspase-3, we have examined the cytotoxic effects of the combined treatment of Ca^2+^ ionophore and CaCl_2_ on neurons (SH-SY5Y cells) and endothelial cells (BAECs). Compared to the untreated control cells, the 10-min treatment with Ca^2+^ ionophore and CaCl_2_ caused the immediate cell death on SH-SY5Y cells on day 1 (Fig. 1). Furthermore, the Ca^2+^ ionophore and CaCl_2_ treatment inhibited BAEC growth through 3-day culture, resulting in 71.4% reduction on day 3 although the Ca^2+^ ionophore and CaCl_2_ treatment did not affect the SH-SY5Y cell proliferation (Fig. 1).

We then investigated whether p5 or p5-primed hADMSCs would protect SH-SY5Y and endothelial cells (BAECs) against the cytotoxicity induced by Ca^2+^ ionophore and CaCl_2_ treatment.

The immediate cytotoxic assay on day 1 and proliferation assay on the following 2 days showed that neither p5 nor CM of untreated hADMSCs affected the SH-SY5Y cells or BAEC numbers with the 10-min treatment of Ca^2+^ ionophore and CaCl_2_, although p5 alone provided the slight protection in SH-SY5Y cells against immediate cytotoxicity induced by Ca^2+^ ionophore and CaCl_2_ treatment (Fig. 1). However, compared with the treatment of Ca^2+^ ionophore and CaCl_2_ alone, SH-SY5Y cell numbers in CM from 100 μg/ml p5-primed hADMSCs collected on day 1 and day 2 and CM from 25 μg/ml p5-primed hADMSCs collected on day 1 significantly increased by 22.8%, 25.3%, and 25.6%, respectively, with immediate cytotoxic assay on day 1. Therefore, the CM of p5-primed hADMSCs provided better protection on neurons than endothelial cells against the immediate cytotoxicity induced by the calpain activation.

More encouragingly, the CM from 100 μg/ml p5-primed hADMSCs completely restored the cell growth in both neurons and endothelial cells after the treatment of Ca^2+^ ionophore and CaCl_2_ with the similar cell numbers as those in the untreated control group on day 3.

### Behavioral Testing

**Fig. 2:**
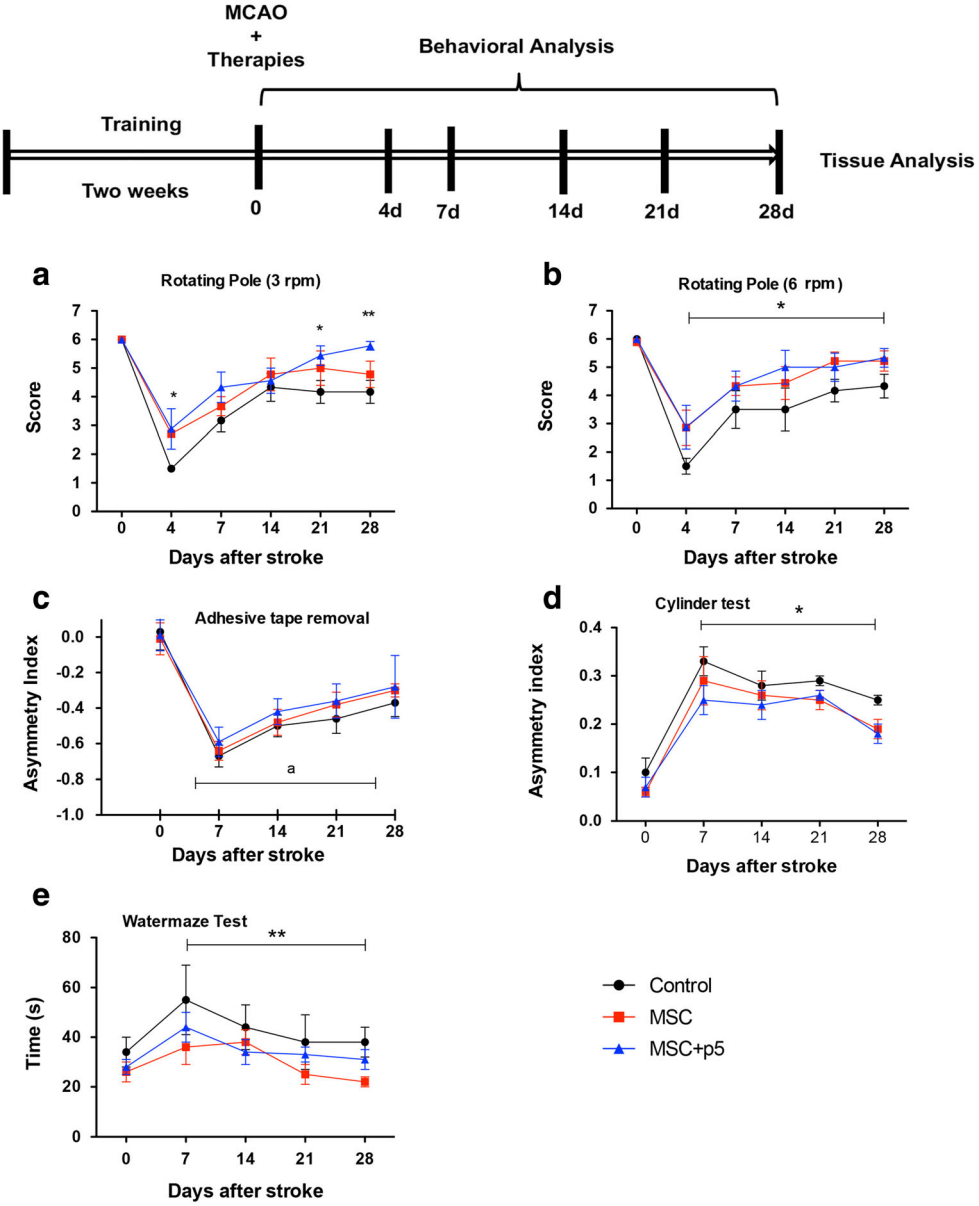
Experimental design (upper panel) and time course of behavioral recovery after treatment with mesenchymal cells and p5. **a**, **b** Functional recovery on a rotating pole at 3 and 6 rpm. **c**, **d** Time course of changes in sensorimotor function recovery after stroke therapy according to adhesive tape removal and cylinder test. Asymmetry of forelimb usage for the postural weight support in rats after stroke therapy. **e** Time course of post-stroke recovery of (spatial) learning and memory by using water maze test. Significance between groups is denoted as *p < 0.05; **p < 0.01, compared to the control group. “a”, significantly different from baseline

The experimental design and time flow of treatment and testing are shown in Fig. 2. To facilitate feeding during the first 3 days post-stroke, all animals were fed with moistened, soft pellets. The mortality rates were almost identical, 14% for each group. All deaths occurred between day 3 and day 10 post-stroke.

### Bilateral Sensorimotor Coordination: Rotating Pole Test

At baseline, rats traversed the rod within 3 s with no mistakes (score of 6) and no differences in performance were observed between rats assigned to control and treatment groups (Fig. 2a). On day 4 following MCAO, for both rotating speeds, the performance on the rotating beam was impaired in all groups but both treatment groups showed significant improvement over the control group (*p* < 0.05) (Fig. 2a, b). Thereafter, the rats treated with the combination MSCs + p5 recovered increasingly better, particularly on day 28 (Fig. 2a) as compared to the control group. However, with increasing difficulty (6 rpm), the significance of recovery of the bilateral sensorimotor coordination diminished (Fig. 2b). In addition, no significant interaction (treatment × duration) effect was found for both speeds in the rotating pole test.

### Asymmetric Sensorimotor Deficit: Adhesive Tape Removal Test

The time course of changes in recovery pattern of control animals, animals treated with MSCs alone, and animals treated with MSCs + p5 is shown in Fig. 2c. The score significantly dropped down from baseline to day 7 and significantly improved on day 21 and day 28 (*p* < 0.001); however, no significant interaction (treatment × duration) was found. Though the scores from the rats treated with the combination of MSCs and p5 were slightly improved over the other treatment or controls at all time points after stroke, they were not statistically significant.

### Asymmetric Sensorimotor Deficit: Cylinder Test

The forelimb asymmetry index indicated a strong deviation to the right (ipsilateral to the injured hemisphere) in controls (0.33 ± 0.04) as compared to MSCs + p5-treated rats (0.25 ± 0.03) on day 7 post-stroke. In addition, both treatment groups showed significant improvement over the control group on day 28 post-stroke (control versus MSCs, control versus MSCs together with peptide; both *p* < 0.01). No significant interaction effect was found between time and treatment factor (Fig. 2d).

### Water Maze Test

The statistical analysis revealed that there was a significant reduction of treatments on time taken by rats to reach the submerged platform (*p* = 0.005). No significant effect of the interaction (time × treatment) was found, suggesting that the pattern of recovery related to spatial learning and memory was similar between different groups at different time points after stroke.

### The Effect of Cell Therapy on the Infarct Volume

**Fig. 3:**
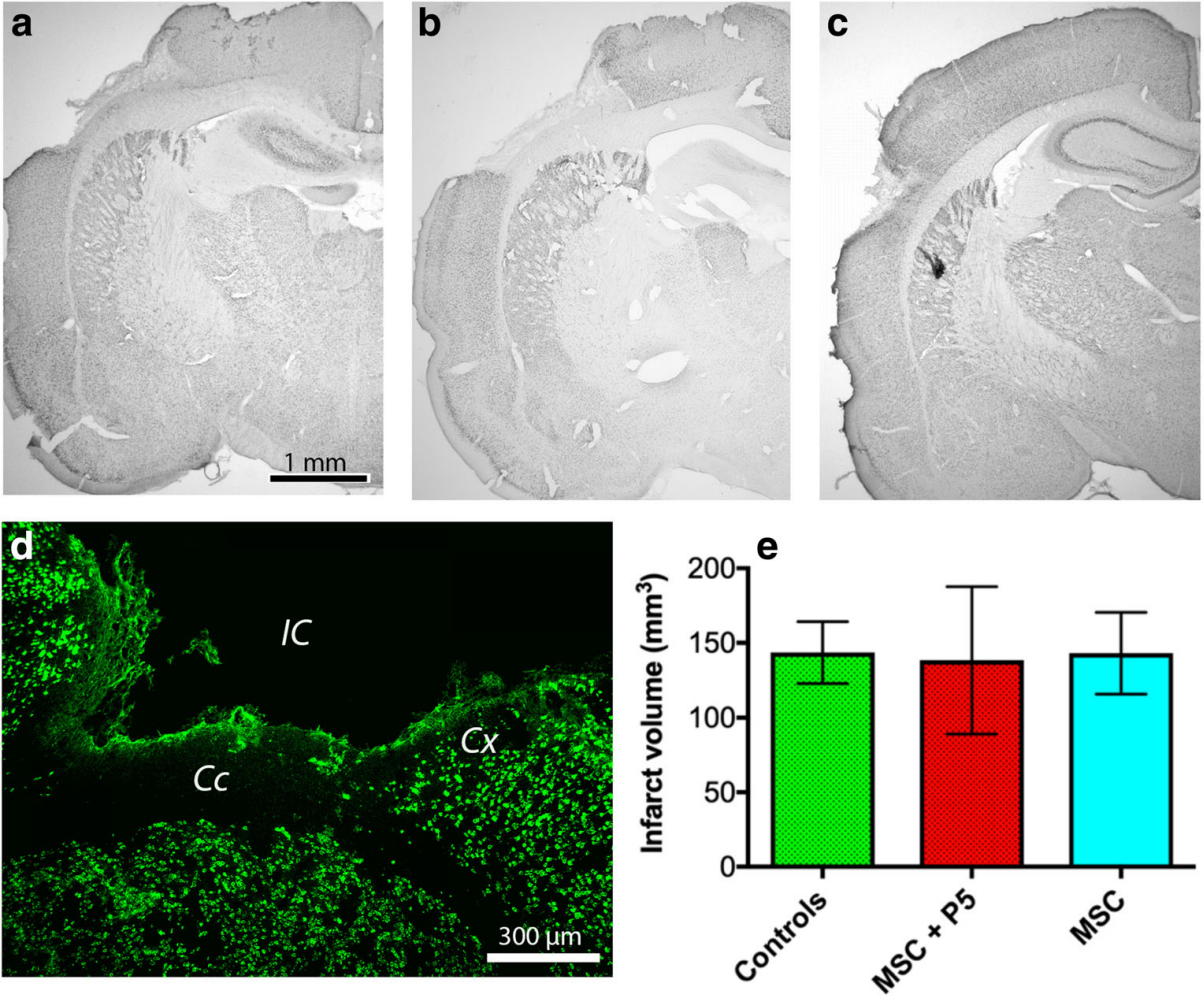
Stroke volumes by NeuN immunohistochemistry. By immunohistochemistry at day 28, the infarct volumes for controls (**a**), MSCs alone (**b**), and MSCs + p5 (**c**) were largely similar (**e**). NeuN immunohistochemistry sharply delineated the infarcted area (**d**). Cc corpus callosum, Cx cortex, IC infarct core

Immunohistochemical staining of the infarct area on day 28 using an anti-NeuN antibody showed that the infarcted cortical volumes were largely similar and independent of treatment as compared to controls (Fig. 3). The anti-NeuN antibody sharply delineated the infarcted area.

### The Survival Rate of Transplanted Cells Was Higher in Animals Treated with hADMSCs + p5

**Fig. 4:**
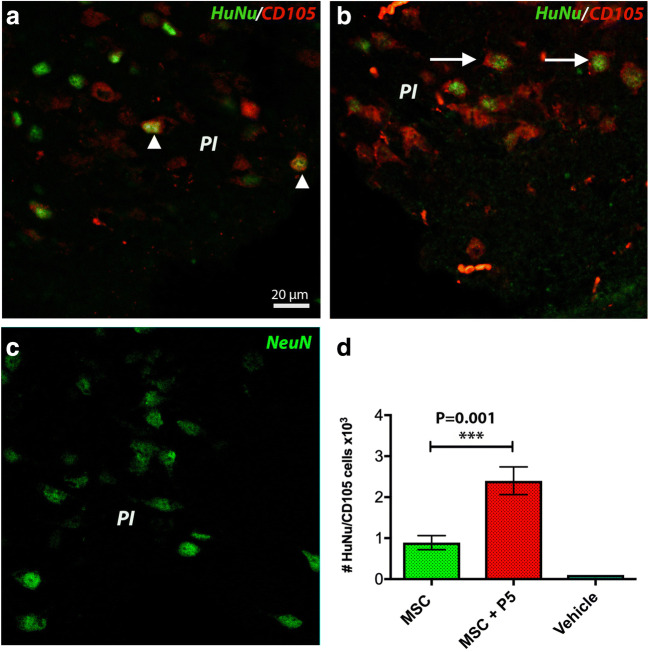
The survival rate of transplanted cells was higher in animals treated with hADMSCs + p5. Note that in the peri-infarcted area of animals treated with hADMSCs + p5 (**b**, arrows), the number of surviving transplanted cells was 2.5-fold higher (**d**) than in hADMSC-treated (**a**) animals. The nuclei in the peri-infarcted area of vehicle-treated animals is shown in **c** (***p = 0.001)

In the ipsilateral hemisphere, the injected human hADMSCs were detected in the perilesional area by double immunofluorescence with anti-human specific antibodies, HuNu (nuclear, green) and CD105 (cytoplasmic, red) (Fig. 4). We noted that in the peri-infarcted area of animals treated with hADMSCs + p5 (Fig. 4b, arrows), the number of surviving transplanted cells was 2.5-fold higher (Fig. 4c) than in hADMSC-treated animals (Fig. 4a).

### hADMSCs Loaded with p5 Reduced the Number of Inflammatory Cells in the Peri-infarcted Area

**Fig. 5:**
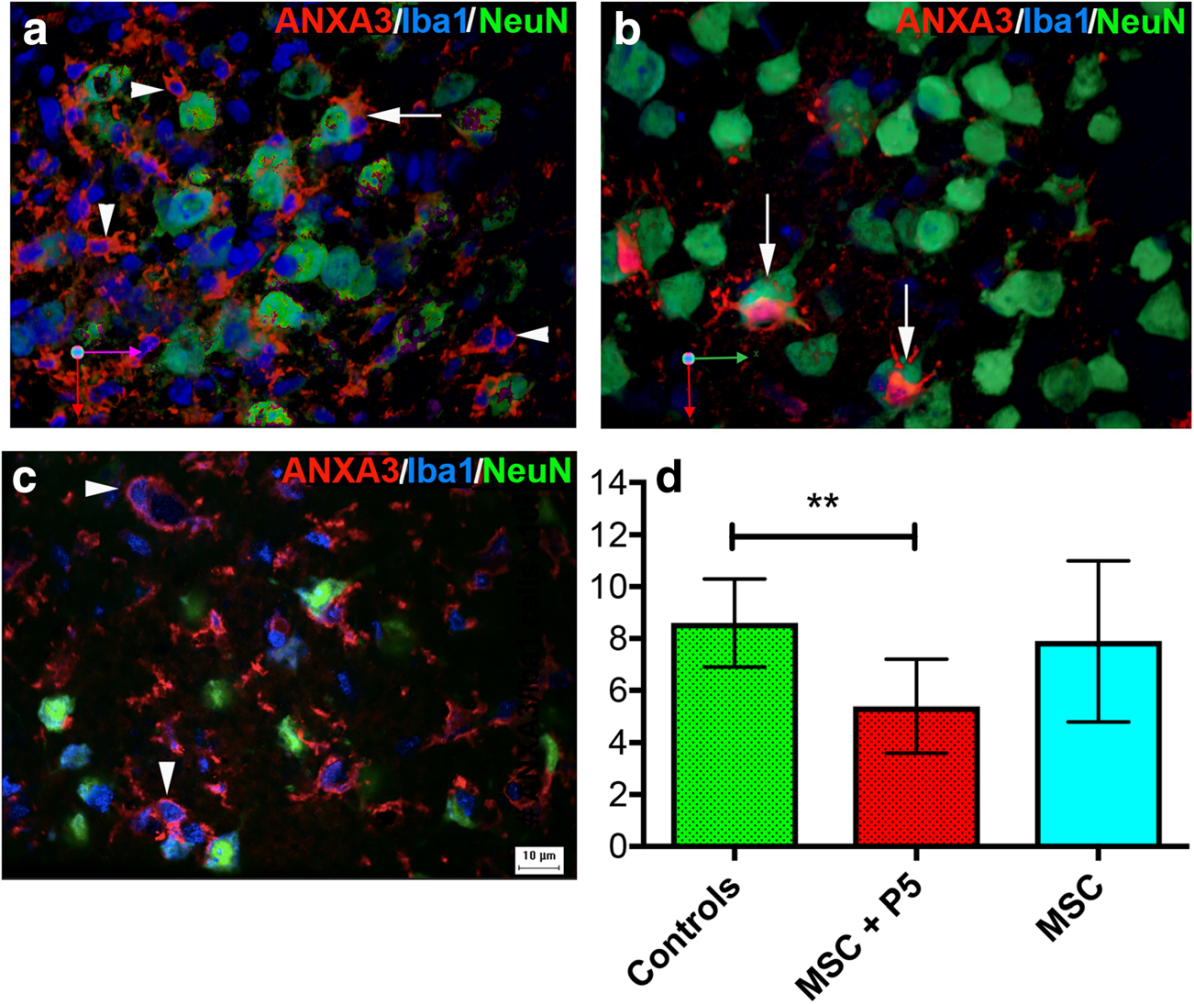
AnnexinA3/NeuN/IbaI triple immunofluorescence phenotyping and cell counting. In animals treated with hADMSCs + p5, the number of co-localized ANXA3/IbaI cells (arrowheads) was decreased by 35% (**d**). Occasionally, microglial cells that are immunopositive for both IbaI and ANXA3 wrapped around neurons (arrows), suggesting an association of ANXA3 expression with a phagocytic phenotype (**a**–**c**) (**p = 0.01)

Cell survival in the perilesional area after stroke is limited by an exaggerated inflammatory response. We asked if the neuroprotective effect of hADMSCs loaded with p5 might be a consequence of decreased number of inflammatory cells in the perilesional area. Indeed, by triple immunofluorescence phenotyping and cell counting, we found that the number of AnxA3/IbaI co-localized cells was decreased by 35% in animals treated with hADMSCs + p5 (Fig. 5d). Occasionally, we found that microglial cells that were immunopositive for both IbaI and ANXA3 wrapped around neurons, highly suggesting an association of ANXA3 expression with a phagocytic phenotype (Fig. 5a–c).

### hADMSCs Loaded with p5 Did Not Increase Vascular Density in the Peri-infarcted Area

**Fig. 6:**
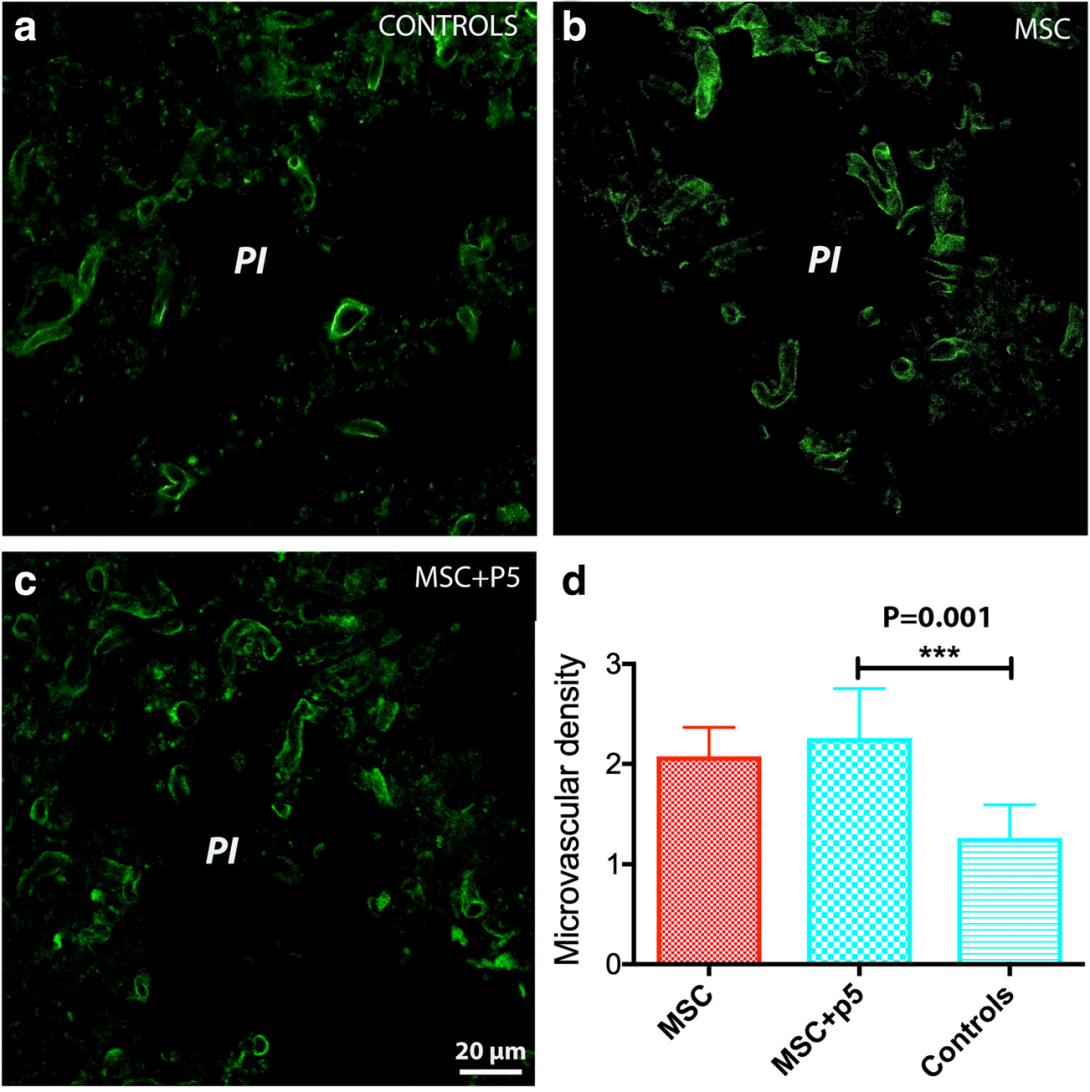
Effect of treatment on post-stroke vascular density. Treatment with hADMSCs alone (**b**) or with hADMSCs + p5 (**c**) led to a significant increase (2.1–2.3-fold) in the vascular density in the peri-infarcted area as compared to control animals (**a**) (***p = 0.001)

Treatment with MSCs stimulates post-stroke angiogenesis [4, 8]. We asked if the neuroprotective effect of hADMSCs loaded with p5 might be a consequence of increased vascularity in the perilesional cortex. We found that the treatment with hADMSCs alone led to a significant increase (2.1-fold) in the vascular density in the peri-infarcted area as compared to control animals (Fig. 6a). Although the treatment of animals with hADMSCs + p5 (Fig. 6b) did also increase (2.3-fold) the vascular density in the peri-infarcted area, the increase was not significant (Fig. 6c, d).

## Discussion

In ischemic stroke, the mechanisms for neuronal cell death has previously been defined in stress states to suggest that an influx of calcium ions into the neurons activates calpain cleavage of p35 into p25 forming a hyperactive complex that induces cell death. Now we report that p5 offers protection to both SH-SY5Y and BAEC cells. In vivo administration of hADMSCs loaded with the therapeutic peptide p5 to post-stroke rats led to improvement in functional recovery and increased number of surviving transplanted cells, most likely by reducing the number of inflammatory ANXA3/IbaI-positive cells in the peri-infarcted region.

Previous work has identified that p5 offers protection to both neurons and endothelial cells [12, 20]. Indeed, this present study has demonstrated a specific protection towards neurons in vitro. We found that p5 offers protection to SH-SY5Y and BAEC cells, although the protection for SH-SY5Y cells was much more significant when compared to the control. Specifically, the data showed p5 protection for SH-SY5Y cells throughout the 3 days in all conditions, peaking on day 2, with continual significance to both control groups. This significant finding indicates the high affinity neurons have for p5. When comparing the protection of BAECs at no time point did p5 offer protection greater than the control group, and similarly to SY5Y cells, protection increased over time until day 2 when p5 25DRD1 and 25DRD2 displayed negative values of cell number. This infers that p5 at that concentration within the endothelial cell inhibited CDK5-p35 rather that CDK5-p25, suggesting that higher dosages are required for BAEC protection.

Surprisingly, the CM from p5-primed hADMSCs provided much stronger protection than p5 alone and CM of untreated hADMSCs did not show any protection against the calpain activation induced by the treatment of Ca^2+^ ionophore and CaCl_2_. These findings suggest that hADMSCs might release other trophic factors, which could work together with released p5 in the CM, thereby providing more potent therapeutic effects.

By immunostaining of the infarct area, the infarcted cortical volumes were largely similar on day 28, suggesting that the treatment with hADMSCs + p5 was not efficacious in reducing infarct volume. However, in the peri-infarcted area of animals treated with hADMSCs + p5, the number of surviving transplanted cells was higher than in hADMSC-treated or control animals, suggesting primarily that the p5 peptide created conditions that supported survival of drug-loaded hADMSCs.

The underlying mechanisms could be three-fold. For one, the therapeutic peptide p5 could block neuronal cell apoptosis through CDK5-p35 and subsequent p53 activation [12]. In addition, the hADMSCs + p5 combination may act by reducing the number of phagocytic ANXA3/IbaI-positive cells in the peri-infarcted region. Indeed, in previous work on experimental stroke, we identified ANXA3 as a novel marker of phagocytic microglia after cerebral ischemia [24]. Therefore, we think that the therapeutic potential of p5 loading of MSCs in the clinic may be exploited in the first week post-stroke when neuroinflammation develops at its maximum and may lead to neuronal death by secondary phagocytosis.

When migrating microglia detect the “eat-me” signals, complete or partial and engulfment of these follows. This process has been coined primary phagocytosis or “phagoptosis” [25, 26]. Further, toxic neuronal insults, such as dying neurons after stroke, may cause an irreversibly exposure of the “eat-me” signal by neurons resulting in phagocytosis of dead neurons or the so-called secondary phagocytosis [27]. It is therefore a challenge to develop stroke therapies that modulate the capability of microglia to phagocytose still viable cells in the injured area after cerebral ischemia [28].

Other supportive mechanisms include stroke-stimulated increase in vascular density after stroke in rats [11]. Using an anti-collagen IV-specific marker, we found that treatment with the hADMSCs is also effective in enhancing the vascular density in the formerly infarcted area of rats during the recovery phase after stroke. However, the hADMSCs + p5 combination did not further increase the post-stroke vascular density and therefore it is unlikely that its beneficial effects on transplanted cell survival in the peri-infarcted area were due the P5 peptide.

In conclusion, administration of human adipose-derived mesenchymal stem cells (hADMSCs) loaded with the therapeutic peptide p5 to post-stroke rats led to improvement in functional recovery and created conditions that supported survival of adipose-derived, drug-loaded MSCs after cerebral ischemia, suggesting its therapeutic potential in patients with stroke and other CNS disorders [28, 29]. In addition, the double immunofluorescence with human cell-specific antibodies revealed that the number of surviving transplanted cells was higher in the peri-infarcted area of animals treated with hADMSCs + p5 than that in hADMSC-treated or control animals, concomitant with reduced number of phagocytic ANXA3/IbaI-positive cells in the peri-infarcted region. However, the mechanisms underlying the beneficial effects of the hADMSCs + p5 combination may not be simple and shall be investigated in a multidisciplinary study. In particular, it is not known whether hADMSCs process p5 into smaller neuroprotective peptides or p5 treatment causes hADMSCs to release neuroprotective factors.

## Electronic Supplementary Material

ESM 1 (DOC 126 kb)
