# Supplementary material for: p5 Peptide-Loaded Human Adipose-Derived Mesenchymal Stem Cells Promote Neurological Recovery After Focal Cerebral Ischemia in a Rat Model
Source: Transl Stroke Res. 2020 May 6;12(1):125–35. doi: 10.1007/s12975-020-00805-0 (PMC7803698; doi:10.1007/s12975-020-00805-0)
Supplement: Supplementary file 1 — (DOC 126 kb) [file 12975_2020_805_MOESM1_ESM.doc]

**Methods**

**Cell culture**

Human adipose derived mesenchymal stem cells (hADMSCs) were provided by Professor Giulio Alessandri and Professor Valentina Ceserani. hAD-MSCs were isolated from peri-umbilical fat tissue and characterized as described [28]. Cells were grown in stem cells medium (SCM) comprised of 80% Iscove’s modified Dulbecco’s medium (IMDM; Sigma-Aldrich) containing 5% fetal bovine serum (FBS; Sigma-Aldrich), 10% NeuroCult medium (Stem Cell Technologies) and 10% endothelial basal medium (EBM) (Lonza) in a humidified incubator with 5% CO_2_ at 37 °C [29]. Human neuroblastoma neuroblastic type SH-SY5Y cells and bovine aortic endothelial cells (BAECs) were cultured in Dulbecco’s modified eagles medium (DMEM; Lonza) supplemented with 10% FBS and 1% L-glutamine.

**p5 Priming of hADMSCs**

p5, a 24-residue peptide, derived from p35, the CDK5 activator, was chemically synthesized with 5-FAM conjugated on the N-terminus and KRKR-KRKR wrapper on the C-terminus [25] (Cambridge Research Biochemicals). The sequence of p5 peptide is [5-FAM]-KEAF-W-DRCLSVINLMSSKMLQINAKRKRKRKR-amide. The toxicity of p5 on hADMSCs was determined in a 24-hour alamarBlue assay (cytotoxicity test; Life Technologies) and in a 3-day alamarBlue assay (anti-proliferative test). Based on these results, the p5 priming of hADMSCs was carried out with two concentrations, 25 µg/ml and 100 µg/ml. Briefly, subconfluent cultures (2 × 10^5^) of hADMSCs were exposed to two dosages of p5. After 24 h of incubation, the cells were washed twice with PBS and trypsinized. Cells were then seeded in a new flask in fresh SCM. After 24 h of culture, the cell conditioned medium (CM) was collected and tested for its protective effects *in vitro* on SH-SY5Y cells and BAECs. The CM from untreated hADMSCs cultured under the same conditions was used as control.

***In vitro* Proliferation Assay**

The protective effect of both p5 or CM of p5 primed hADMSCs on SH-SY5Y cells and BAECs proliferation after the treatment of Ca^2+^ ionophore combined with CaCl_2_ was determined by the alamarBlue assay (Life Technologies). Briefly, 7.0 × 10^4^ SH-SY5Y cells or 1.0 × 10^3^ BAECs were plated in 96-well multiwell plates in DMEM. The cells were then treated with 5 µM calcium ionophore A23187 (Sigma-Aldrich) and 2.5 mM CaCl_2_ (Sigma-Aldrich) with or without p5 or CM of p5-primed hADMSCs. Three wells were devoted for the assay every day for 3 days, and time-course of cell proliferation was monitored. In practice, 10 µl of the alamarBlue reagent (Life Technologies) was added to each well and the plates were incubated at 37 °C for 4 h in a humidified atmosphere with 5% CO_2_ before recording fluorescence (530_Ex_/590_Em_) using a Synergy HT multi-detection microplate reader (BioTek Instruments). According to the standard curves for SH-SY5Y cells and BAECs generated from fluorescence signals plotted against the number of cells (between 0 and 1.6 × 10^5^ SH-SY5Y cells and between 0 and 2.0 × 10^4^ BAECs, respectively), cell numbers were calculated from the fluorescence signals.

**Animals and Experimental Design**

The subjects of these experiments were male Sprague-Dawley rats (*n =* 40; 3-4 months of age) 320-400 g kept under standard laboratory conditions with free access to food and water. The numbers reported in the results refer to the number of animals that survived the surgery and completed the 4-week testing period. All experiments were approved by the Institutional Animal Care and Use Committee of the Medical University of Craiova.

**Randomization**

A scientist was in charge of randomization by (i) group assignment, (ii) behavioral testing; (iii) surgery assignment; and (iv) treatment assignment.

Behavioral Testing

To evaluate changes in neurological function associated with ischemia, the rats were subjected to a variety of somatosensory, motor, learning, and memory tests before and after surgery. All testing was performed from 09:00 to 11:00. Results obtained before surgery were used to define 100% functionality for each animal on each test, and functional recovery was expressed as percent recovery relative to the pre-surgery baseline.

Bilateral Sensorimotor Coordination: Rotating Beam-walking Test

The rotating pole task assesses coordination and sensorimotor function in the MCAO model. Each rat was tested for its ability to cross a rotating (6 rpm) horizontal rod. The score assessment was done as previously described [30]. Briefly, the time taken for the rat to traverse the rotating cylinder and join a group of rats visible at the finish line was measured. The score assessment was twofold: (i) time (seconds) required to traverse the rotating cylinder and (ii) the score as follows: 0 = rat falls immediately (onto a soft surface); 1 = rat does not walk forward, but stays on the rotarod; 2 = rat walks, but falls before reaching the goal; 3 = rat traverses the rod successfully, but the limbs are used asymmetrically; 4 = the left hindlimb is used less than 50% of the time taken to traverse the rod; 5 = the rat successfully traverses the rod, but with some difficulties; 6 = no mistakes, symmetric movements.

**Asymmetric Sensorimotor Deficit: Adhesive Tape Removal Test (Tape Test) and Cylinder Test**

We assessed the asymmetry of sensorimotor deficit of the forelimbs induced by unilateral MCAO, by tape-test and cylinder test. During the procedures, the rats were placed in a glass cylinder and videotaped from below. The recordings were scored using slow-motion video. For the tape test, sticky patches were applied on the distal hairless parts of the forelimbs and the removal time from both limbs was measured. Three trials were done separately for each limb and the means of the values were noted. If the animal did not remove the tape within 180 s, the timer was stopped. For the cylinder test, the forelimb use asymmetry during vertical exploration of the walls of a 20 cm diameter and 40 cm height glass cylinder was measured. The rat was videotaped for 3-10 min, depending on the degree of movement during the trial. If the animal showed signs of inactivity, rearing was encouraged by wafting odors above the cylinder opening, making gentle sounds. Number of contacts between each limb and the walls of the cylinder were counted separately. Rats which did not make at least 20 exploratory contacts with the walls were excluded from the analysis procedure. The asymmetry index was calculated as (Right − Left)/(Right + Left), where Right and Left are the number of contacts of the right.

Spatial Learning and Memory: Morris Water Maze

The Morris water maze task was used to assess spatial learning and memory. One week before surgery, aged rats were trained to find a submerged platform in a large (180 cm-diameter) pool filled to within 20 cm of the upper edge with water maintained at 26 °C. The pool was divided into four compass quadrants (north, south, east, and west). Several visual stimuli were placed in each of the four quadrants. For the acquisition of spatial learning, each animal underwent a block of four trials per day for 7 days. Before the first trial, the rat was placed on the hidden platform for 30 s by the investigator. Each trial consisted of placing the rat in the water at one of the randomly selected four starting locations around the pool perimeter. Each rat was allowed a maximum of 60 s to find the hidden platform and remain on it for 30 s. If a rat failed to find the platform within 60 s, the rat was placed on the platform for 30 s by the investigator. The time and distance required to find the hid- den platform during these four acquisition trials were averaged [31] . The swim path was recorded by an image analysis system (Noldus, Holland) that computed path length and percentage of time spent in each of the four quadrants. Functional restoration of spatial learning and memory was estimated by weekly testing after MCAO and in total for 8 weeks.

**Surgery**

18 h prior to surgery, rats were deprived of food to minimize variability in ischemic damage that can result from varying plasma glucose levels. Water remained available at all times. In all cases, surgery was performed between 09:00 and 13:00. Cerebral infarction was induced by transcranial interruption of blood flow by transiently lifting the middle cerebral artery (MCA) with a tungsten hook as previously described [32]**.** Throughout surgery, anesthesia was maintained by spontaneous inhalation of 2% isoflurane in a mixture of 70% nitrous oxide and 30% oxygen. Body temperature was maintained at 37 °C by a Homeothermic Blanket System (Harvard Apparatus). The local changes in blood flow were monitored using a laser Doppler device (Perimed, Stockholm, Sweden). A decrease in the laser Doppler signal to *<* 20% of control values was considered to indicate successful MCA occlusion. After 90 min, the tungsten hook was released and the common carotid arteries were re-opened. Surgery was performed under antiseptic conditions to minimize the risk of infection. The rats then received 0.05 mg/kg SC buprenorphine (sc) for treatment of postoperative pain.

Subsequent to survival times of 28 days, the rats were deeply anesthetized with 1.5% isoflurane in 75% nitrous oxide and 25% oxygen, and perfused with neutral buffered saline followed by buffered 4% freshly depolymerized paraformaldehyde. The brain was removed, post-fixed in 4% buffered paraformaldehyde for 24 h, cryoprotected in 15% glycerol prepared in 10 mM phosphate buffered saline, flash-frozen in isopentane, and stored at −70 °C until sectioning.

Treatments

The contents of the frozen vials containing hADMSCs are re-suspended in a T75 flask containing 10 ml of stem cells medium (SCM). After a period of about 24 h, once the cells have settled on the bottom of the flask, the media was removed and replaced with fresh SCM primed with the therapeutic peptide P5 at a final concentration of 10 µg/ml using the minimum amount of media required i.e. in a T25 flask, 5 ml. The cells were then further incubated for 24 h. After the incubation period, the cells were washed 3 times with PBS before trypsinization. One group of rats (*n* = 8) was treated with 1 × 10^6^ hADMSCs, given intracortically at 3 different locations in the exposed area proximal to the occluded middle cerebral artery, immediately after stroke. The second group (*n* = 8) was treated with hADMSCs + P5 and the control (*n* = 8) group was treated with the vehicle.

Determination of Infarct Volume by Immunohistostaining

To assess the size of the infarct induced by focal ischemia, we used mouse anti-NeuN immunostaining [33]. Every 20th free-floating section of 25 µm was immunostained for NeuN to cover the entire infarcted volume, which was then calculated as the sum of the partial areas using ImageJ. Briefly, the tissue was incubated with a mouse anti-NeuN (1:1000, Millipore, Germany) at 4 °C overnight. For the chemical staining, the next day sections were washed with 1xPBS/0.2% Tween and incubated with the Histofine polymer, goat-anti mouse-IgG-POD (Nichirei, Japan) for another 20 hrs followed by staining with 3,3’-diaminobenzidine. After drying overnight, sections were mounted under a coverslip in DPX. For fluoresecence staining, sections were rinsed with PBS and incubated with Alexa Fluor® 488 goat anti-mouse IgG. After a final rinsing, sections were brought to Superfrost Plus slides and mounted using PVA/DABCO-containing medium. Integration of the resulting partial volumes (partial areas × number of sections × section thickness × section intervals) yielded the total volume of the infarct as previously described [32,33].

**Immunofluorescence**

Collagen IV immunofluorescence

Sections (25 µm-thick) were cut on a freezing microtome and processed for immunohistochemistry as previously described [34]. Briefly, after incubation with blocking solutions containing 3% donkey serum/10 mmol/L PBS/0.3% Tween 20, tissue sections were exposed overnight at 4°C to rabbit anti-collagen IV (1:2000, abcam, UK) diluted in PBS containing 3% normal donkey serum and 0.3% Tween 20. After washing in PBS containing 0.3% Tween, the signal was amplified utilizing an anti-rabbit polymer-based secondary detection system (Histofine polymer-HRP, Nichirei, Japan) diluted 1:10 in PBS containing 1% normal goat serum and 0.3% Tween 20. After washing in PBS, sections were stained with tyramide-FITC as previously described [34].

HuNu/CD105 double immunofluorescence

Cryostat, free-floating sections of 25 µm were fixed in 4% paraformaldehyde for 15 min and then washed extensively with PBS and processed using an automatic staining machine for floating sections, Tingomat 501 (http://tingomat.com/imprint-impressum/). For phenotyping of transplanted cells, the tissue was incubated with a mix of rabbit anti-mouse HuNu (1:1000, Novus Biologicals, UK) and mouse anti-human CD105 (1:1000, antibodies-on-line, Aachen, Germany) at 4 °C overnight. Next day, sections were rinsed with PBS and incubated with a mix of Alexa Fluor® 488 goat anti-rabbit IgG and Alexa Fluor® 555 goat anti-mouse IgG for CD105. After final rinsing, sections were brought to Superfrost Plus slides and mounted using PVA/DABCO-containing medium.

Annexin A3/NeuN/Iba1 triple immunofluorescence

Cryostat, free-floating sections of 25 µm were fixed in 4% paraformaldehyde for 15 min and then washed extensively with PBS and incubated serially with the following primary antibodies, rabbit anti-AnxA3 (1:500, abcam, UK), goat anti-Iba1 (1:3000, abcam, UK) and mouse anti-NeuN (1:1000, Millipore, Germany). Secondary reagents were donkey anti-mouse Alexa 488, donkey anti rabbit-Alexa 568 and donkey anti goat-Alexa 647.

**Cell Counting of Co-localized Cells**

 The number of labeled cells at the reperfusion times of 28 days was determined by counting cells on every tenth section in systematic random series across the entire infarcted volume. To this end, a sequence of confocal counting images of 161x242x25 µm, spaced 0.1 µm apart across a 25 µm-thick section and covering 30% of the infarcted area, was taken for fluorescently labeled cells. An integration of (HuNu + CD105-positive) cells was achieved by multiplying the number of cells per section times 3.3 (the counting boxes that were quantitated covered one-third of the area of each section) times the section interval [32]. The number of co-labeled AnxA3/Iba1 cell was counted using a similar approach.

**Quantitation of Microvascular Density**

Microvascular density was quantitated using the “hot spot” analysis. Briefly, hot-spots, i.e. regions with a high density of collagen IV-positive microvessels, were identified using a 40x objective and were then counted using 20x objective, corresponding to a microscopic field of 0.7386 mm^2^ as previously described [34]. Counting was done by two independent observers and the results are expressed as means±SD.

**Microscopy**

For light microscopy, a Nikon Eclipse (Nikon, Duesseldorf, Germany) was used. Confocal microscopy images were acquired using a Zeiss LSM710 laser-scanning confocal system with spectral detection capabilities, and Zen 2010 software version 6.0 (Carl Zeiss Microscopy GmbH, Jena, Germany) was used for image acquisition and analysis. Excitation light was provided by 488, 543, and 634 nm laser lines; fluorescence emission was detected at 500-530 nm for FITC (green), 550-600 nm for rhodamin (red) and 650-710 nm for Cy5 (blue) in separate tracks, using a confocal aperture of 1 Airy unit. Some of the images were acquired as *z* -stacks and a 3D reconstruction was performed using Image-J.

**Statistical Analyses**

Statistical analysis was performed using SPSS (V 22.0). Behavioral data were analyzed using two-way ANOVA (treatment × duration) with post-stroke time as a repeated measurement factor. The factor treatment had 3 levels (control, MSCs and MSCs + p5) and the factor duration had 5 levels (0: before stroke, 7, 14, 21, 28 days after stroke). Only in the rotating pole test, there were 6 levels, where one more time point of measurement (day 4) was included. The Greenhouse-Geisser correction was applied where the sphericity was violated. T-tests were performed for the single parameter comparison, and unequal variance between groups was assumed. All values are presented as mean ± SEM. p values < 0.05 were considered statistically significant.

**References**

1. Kopen GC, Prockop DJ, Phinney DG. Marrow stromal cells migrate throughout forebrain and cerebellum, and they differentiate into astrocytes after injection into neonatal mouse brains. Proc Natl Acad Sci U S A 1999;96:10711-6. http://doi.org/[10.1073/pnas.96.19.10711](https://doi.org/10.1073/pnas.96.19.10711)

2. Chen JR, Cheng GY, Sheu CC, Tseng GF, Wang TJ, Huang YS. Transplanted bone marrow stromal cells migrate, differentiate and improve motor function in rats with experimentally induced cerebral stroke. J Anat 2008;213:249-58. http://doi.org/[10.1111/j.1469-7580.2008.00948.x](https://doi.org/10.1111/j.1469-7580.2008.00948.x)

3. Chen J, Li Y, Katakowski M, et al. Intravenous bone marrow stromal cell therapy reduces apoptosis and promotes endogenous cell proliferation after stroke in female rat. J Neurosci Res 2003;73:778-86. http://doi.org/[10.1002/jnr.10691](https://doi.org/10.1002/jnr.10691)

4. Honmou O, Onodera R, Sasaki M, Waxman SG, Kocsis JD. Mesenchymal stem cells: therapeutic outlook for stroke. Trends Mol Med 2012;18:292-7. http://doi.org/[10.1016/j.molmed.2012.02.003](https://doi.org/10.1016/j.molmed.2012.02.003)

5. Shichinohe H, Ishihara T, Takahashi K, Tanaka Y, Miyamoto M, Yamauchi T et al. Bone marrow stromal cells rescue ischemic brain by trophic effects and phenotypic change toward neural cells.Neurorehabil Neural Repair. 2015; 29(1):80-9. <http://doi.org/10.1177/1545968314525856>

6. Bi M, Wang J, Zhang Y, Li L, Wang L, Yao R, et al. Bone mesenchymal stem cells transplantation combined with mild hypothermia improves the prognosis of cerebral ischemia in rats. PLoS One 2018;13. http://doi.org/[10.1371/journal.pone.0197405](https://doi.org/10.1371/journal.pone.0197405)

7. Guo F, Lv S, Lou Y, et al. Bone marrow stromal cells enhance the angiogenesis in ischaemic cortex after stroke: involvement of notch signalling. Cell Biol Int 2012;36:997-1004. http://doi.org/[10.1042/CBI20110596](https://doi.org/10.1042/CBI20110596)

8. Tate CC, Fonck C, McGrogan M, Case CC. Human mesenchymal stromal cells and their derivative, SB623 cells, rescue neural cells via trophic support following in vitro ischemia. Cell Transplant 2010;19:973-84. http://doi.org/[10.3727/096368910X494885](https://doi.org/10.3727/096368910X494885)

9. Leu S, Lin YC, Yuen CM, et al. Adipose-derived mesenchymal stem cells markedly attenuate brain infarct size and improve neurological function in rats. J Transl Med 2010;8:63. http://doi.org/[10.1186/1479-5876-8-63](https://doi.org/10.1186/1479-5876-8-63)

10. Li G, Yu F, Lei T, et al. Bone marrow mesenchymal stem cell therapy in ischemic stroke: mechanisms of action and treatment optimization strategies. Neural Regen Res 2016;11:1015-24. http://doi.org/[10.4103/1673-5374.184506](https://doi.org/10.4103/1673-5374.184506)

11. Deng YB, Ye WB, Hu ZZ, et al. Intravenously administered BMSCs reduce neuronal apoptosis and promote neuronal proliferation through the release of VEGF after stroke in rats. Neurol Res 2010;32:148-56. http://doi.org/[10.1179/174313209X414434](https://doi.org/10.1179/174313209X414434)

12. Balseanu AT, Buga AM, Catalin B, et al. Multimodal approaches for regenerative stroke therapies: combination of granulocyte colony-stimulating factor with bone marrow mesenchymal stem cells is not superior to G-CSF alone. Front Aging Neurosci 2014;6:130. http://doi.org/[10.3389/fnagi.2014.00130](https://doi.org/10.3389/fnagi.2014.00130).

1. Slevin M, Krupinski J. [Cyclin-dependent kinase-5 targeting for ischaemic stroke](http://www.ncbi.nlm.nih.gov/pubmed/18983942). Curr Opin Pharmacol 2009; 9:119-24. http://doi.org/[10.1016/j.coph.2008.10.003](https://doi.org/10.1016/j.coph.2008.10.003).
2. Kusakawa G, [Saito](http://www.ncbi.nlm.nih.gov/pubmed/?term=Saito T%5BAuthor%5D&cauthor=true&cauthor_uid=10748088) T, [Onuki](http://www.ncbi.nlm.nih.gov/pubmed/?term=Onuki R%5BAuthor%5D&cauthor=true&cauthor_uid=10748088) R, et al. Calpain-dependent proteolytic cleavage of the p35 cyclin-dependent kinase 5 activator to p25. J Biol Chem 2000; [275:](http://www.ncbi.nlm.nih.gov/pubmed/?term=Calpain-dependent+Proteolytic+Cleavage+of+the+p35)17166-72. http://doi.org/[10.1074/jbc.M907757199](https://doi.org/10.1074/jbc.M907757199).
3. Lee MS, Kwon YT, Li M, Peng J, Friedlander RM, Tsai LH. Neurotoxicity induces cleavage of p35 to p25 by calpain. Nature 2000; 405:360-4. http://doi.org/[10.1038/35012636](https://doi.org/10.1038/35012636).
4. Zheng YL, Li C, Hu YF, et al. Cdk5 inhibitory peptide (CIP) inhibits Cdk5/p25 activity induced by high glucose in pancreatic beta cells and recovers insulin secretion from p25 damage. PLoS One 2013;8:e63332. http://doi.org/[10.1371/journal.pone.0063332](https://doi.org/10.1371/journal.pone.0063332).
5. Kesavapany S, Zheng YL, Amin N, Pant HC. Peptides derived from Cdk5 activator p35, specifically inhibit deregulated activity of Cdk5. Biotechnol J 2007;2:978-87. http://doi.org/[10.1002/biot.200700057](https://doi.org/10.1002/biot.200700057).
6. Zhang L, Liu W, Szumlinski KK, Lew J. p10, the N-terminal domain of p35, protects against CDK5/p25-induced neurotoxicity. Proc Natl Acad Sci USA 2012;109:20041-6. http://doi.org/[10.1073/pnas.1212914109](https://doi.org/10.1073/pnas.1212914109).
7. Tan X, Chen Y, Li J, et al. The inhibition of Cdk5 activity after hypoxia/ischemia injury reduces infarct size and promotes functional recovery in neonatal rats. Neuroscience 2015;290:552-60.

http://doi.org/[10.1016/j.neuroscience.2015.01.054](https://doi.org/10.1016/j.neuroscience.2015.01.054).

1. Binukumar BK, Zheng YL, Shukla V, Amin ND, Grant P, Pant HC. TFP5, a peptide derived from p35, a Cdk5 neuronal activator, rescues cortical neurons from glucose toxicity. J Alzheimers Dis 2014;39(4):899-909. http://doi.org/ [10.3233/JAD-131784](https://doi.org/10.3233/JAD-131784).
2. Shukla V, Zheng YL, Mishra SK, et al. A truncated peptide from p35, a Cdk5 activator, prevents Alzheimer's disease phenotypes in model mice. FASEB J 2013;27:174-86. http://doi.org/[10.1096/fj.12-217497](https://doi.org/10.1096/fj.12-217497).
3. Zheng YL, Amin ND, Hu YF, et al. A 24-residue peptide (p5), derived from p35, the Cdk5 neuronal activator, specifically inhibits Cdk5-p25 hyperactivity and tau hyperphosphorylation. J Biol Chem 2010;285:34202-12. http://doi.org/[10.1074/jbc.M110.134643](https://doi.org/10.1074/jbc.M110.134643).
4. 23. Li Z, Fan D, Xiong D. Mesenchymal stem cells as delivery vectors for anti-tumor therapy. Stem Cell Investig 2015;2:6. http://doi.org/0.39782015 Mar 26;2:6. doi: 10.3978.
5. Nava S, Sordi V, Pascucci L, Tremolada C, Ciusani E, Zeira O, et al. Long-Lasting Anti-Inflammatory Activity of Human Microfragmented Adipose Tissue. Stem Cells Int. 2019;2019:5901479. http://doi.org/10.1155/2019/5901479. eCollection 2019

25. Lee J-H, Kim H-S, Lee S-J, Kim K-T. Stabilization and activation of p53 induced by Cdk5 contributes to neuronal cell death. Journal of Cell Science. 2007;120:2259–71. http://doi.org/ [10.1242/jcs.03468](https://doi.org/10.1242/jcs.03468)

26. Iyirhiaro GO, Im DS, Boonying W, Callaghan SM, During MJ, Slack RS, et al. Cdc25A Is a Critical Mediator of Ischemic Neuronal Death In Vitro and In Vivo. J Neurosci. 2017;37:6729–40. http://doi.org/ [10.1523/JNEUROSCI.3017-16.2017](https://doi.org/10.1523/JNEUROSCI.3017-16.2017)

27. Pacioni S, D’Alessandris QG, Giannetti S, Morgante L, De Pascalis I, Coccè V, et al. Mesenchymal stromal cells loaded with paclitaxel induce cytotoxic damage in glioblastoma brain xenografts. Stem Cell Res Ther. 2015;6:194. http://doi.org/ [10.1186/s13287-015-0185-z](https://doi.org/10.1186/s13287-015-0185-z)

28. Fang W-H, Kumar S, McDowell G, Smith D, Krupinski J, Olah P, et al. Mesenchymal Stem Cells Loaded with p5, Derived from CDK5 Activator p35, Inhibit Calcium-Induced CDK5 Activation in Endothelial Cells. Stem Cells Int. 2016;2016:2165462. http://doi.org/ [10.1155/2016/2165462](https://doi.org/10.1155/2016/2165462)

29. Buchhold B, Mogoanta L, Suofu Y, Hamm A, Walker L, Kessler C, et al. Environmental enrichment improves functional and neuropathological indices following stroke in young and aged rats. Restor Neurol Neurosci. 2007;25:467–84.

30. Tottori K, Nakai M, Uwahodo Y, Miwa T, Yamada S, Oshiro Y, et al. Attenuation of scopolamine-induced and age-associated memory impairments by the sigma and 5-hydroxytryptamine(1A) receptor agonist OPC-14523 (1-[3-[4-(3-chlorophenyl)-1-piperazinyl]propyl]-5-methoxy-3,4-dihydro-2[1H]-quinolinone monomethanesulfonate). J Pharmacol Exp Ther. 2002;301:249–57. [10.1124/jpet.301.1.249](https://doi.org/10.1124/jpet.301.1.249)

31. Popa-Wagner A, Stöcker K, Balseanu AT, Rogalewski A, Diederich K, Minnerup J, et al. Effects of granulocyte-colony stimulating factor after stroke in aged rats. Stroke. 2010;41:1027–31. http://doi.org/ [10.1161/STROKEAHA.109.575621](https://doi.org/10.1161/STROKEAHA.109.575621)

32. Popa-Wagner A, Badan I, Walker L, Groppa S, Patrana N, Kessler C. Accelerated infarct development, cytogenesis and apoptosis following transient cerebral ischemia in aged rats. Acta Neuropathol. 2007;113:277–93. http://doi.org/ [10.1007/s00401-006-0164-7](https://doi.org/10.1007/s00401-006-0164-7)

33. Buga AM, Margaritescu C, Scholz CJ, Radu E, Zelenak C, Popa-Wagner A. Transcriptomics of post-stroke angiogenesis in the aged brain. Front Aging Neurosci. 2014;6:44. http://doi.org/ [10.3389/fnagi.2014.00044](https://doi.org/10.3389/fnagi.2014.00044).

34. Kim JY, Kawabori M, Yenari MA. Innate inflammatory responses in stroke: mechanisms and potential therapeutic targets. Curr Med Chem. 2014;21:2076–97. http://doi.org/ [10.2174/0929867321666131228205146](https://doi.org/10.2174/0929867321666131228205146)

35. Liu K, Guo L, Zhou Z, Pan M, Yan C. Mesenchymal stem cells transfer mitochondria into cerebral microvasculature and promote recovery from ischemic stroke. Microvasc Res. 2019;123:74–80. http://doi.org/ [10.1016/j.mvr.2019.01.001](https://doi.org/10.1016/j.mvr.2019.01.001)

36. Nam HS, Kwon I, Lee BH, Kim H, Kim J, An S, et al. Effects of Mesenchymal Stem Cell Treatment on the Expression of Matrix Metalloproteinases and Angiogenesis during Ischemic Stroke Recovery. PLoS ONE. 2015;10:e0144218. http://doi.org/10.1371/journal.pone.0144218

37. Junker H, Suofu Y, Venz S, Sascau M, Herndon JG, Kessler C, et al. Proteomic identification of an upregulated isoform of annexin A3 in the rat brain following reversible cerebral ischemia. Glia. 2007;55:1630–7.

38. Fuhrmann M, Bittner T, Jung CKE, Burgold S, Page RM, Mitteregger G, et al. Microglial Cx3cr1 knockout prevents neuron loss in a mouse model of Alzheimer’s disease. Nat Neurosci. 2010;13:411–3. http://doi.org/ [10.1002/glia.20581](https://doi.org/10.1002/glia.20581)

39. Neher JJ, Neniskyte U, Zhao J-W, Bal-Price A, Tolkovsky AM, Brown GC. Inhibition of microglial phagocytosis is sufficient to prevent inflammatory neuronal death. J Immunol. 2011;186:4973–83. http://doi.org/ [10.4049/jimmunol.1003600](https://doi.org/10.4049/jimmunol.1003600)

40. Brown GC, Neher JJ. Microglial phagocytosis of live neurons. Nat Rev Neurosci. 2014;15:209–16. http://doi.org/ [10.1038/nrn3710](https://doi.org/10.1038/nrn3710)

41. Stonesifer C, Corey S, Ghanekar S, Diamandis Z, Acosta SA, Borlongan CV. Stem cell therapy for abrogating stroke-induced neuroinflammation and relevant secondary cell death mechanisms. Prog Neurobiol. 2017;158:94–131. http://doi.org/ [10.1016/j.pneurobio.2017.07.004](https://doi.org/10.1016/j.pneurobio.2017.07.004)

42. Lee J-Y, Lin R, Nguyen H, Grant Liska M, Lippert T, Kaneko Y, et al. Histopathological and Behavioral Assessments of Aging Effects on Stem Cell Transplants in an Experimental Traumatic Brain Injury. Methods Mol Biol. 2019;2045:299–310. http://doi.org/ [10.1007/7651_2018_121](https://doi.org/10.1007/7651_2018_121)
